# Preparation and characterization of an intelligent film based on fish gelatin and *Coleus scutellarioides* anthocyanin to monitor the freshness of rainbow trout fish fillet

**DOI:** 10.1002/fsn3.3068

**Published:** 2022-09-21

**Authors:** Fahimeh Hematian, Homa Baghaei, Abdorreza Mohammadi Nafchi, Marzieh Bolandi

**Affiliations:** ^1^ Department of Food Science and Technology, Damghan Branch Islamic Azad University Damghan Iran; ^2^ Food Technology Division, School of Industrial Technology Universiti Sains Malaysia Penang Malaysia; ^3^ Green Biopolymer, Coatings & Packaging Cluster, School of Industrial Technology Universiti Sains Malaysia Penang Malaysia

**Keywords:** anthocyanin, intelligent packaging, pH sensing, release kinetics, TVB‐N

## Abstract

In this study, a pH‐sensitive indicator based on fish gelatin and *Coleus scutellarioides* anthocyanin extract (CSAE) was prepared and characterized. Films were prepared using the solvent casting method and different levels of CSAE, including 10 ml (CSG1), 20 ml (CSG2), and 30 ml (CSG3), and 0 ml (CSG0) as a control sample. The mechanical, optical, and pH sensing of active films and the release of anthocyanins from the films were investigated. The relationship between the total volatile basic nitrogen (TVB‐N) of fish fillets and a* color index of films was studied. By incorporation of CSAE, the flexibility of films increased, while the tensile strength and UV–Vis light transmittance through the films decreased (*p* < .05). The films containing the CSAE had a darker, yellowish, and reddish color than the control film. There was a significant relationship between the pH variation and the film color. The films had a purple color at acidic pH, and their color changed to green at an alkaline pH, indicating the sensitivity of the produced films to pH changes. There was a significant relationship between the TVB‐N value of fish fillets and the a* index of the film during the 16 h storage time. The results showed that by increasing TVB‐N values of the fillets, the a* color index decreased, and the films' color changed from purple to colorless. In summary, the active films prepared with fish gelatin and CSAE could be used as pH‐sensitive intelligent packaging to display the freshness of fishery products.

## Graphical abstract

A pH‐sensitive indicator based on fish gelatin and *Coleus scutellarioides* anthocyanin extract (CSAE) was prepared and characterized. There was also a significant relationship between the TVB‐N content of fish fillets and the a* index of the film during the 16 hours storage period so that with the increase of TVB‐N values of the fillets during the storage, the a* color index decreased and the color of the films changed from purple to colorless.

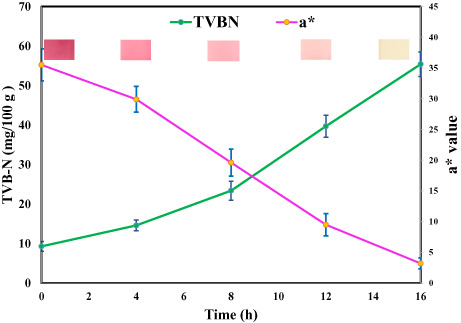

## 1 INTRODUCTION

Seafood is a highly perishable food product because the action of internal enzymes strongly influences its quality, chemical reactions, microbial growth, and lipid oxidation (Volpe et al., 2019; Yu et al., 2020). In addition, due to the action of microorganisms on seafood, a wide range of volatile compounds are produced. As a result of the accumulation of these compounds in the product, an unpleasant odor and taste are created, and the product becomes unacceptable to the consumer (Zhuang et al., 2021). Therefore, the freshness of seafood products is one of the keys and significant factors for consumers, so in recent years, intelligent food packaging has been developed to monitor the freshness of these products using biopolymer films and natural pigments (Ekramian et al., 2021; Gasti et al., 2021).

The use of detectors in a smart package is one of the remarkable cases in the seafood and meat industry (Choi et al., 2017; Esfahani et al., 2022). Using smart or intelligent packaging and indicators, consumers can evaluate the quality of products in terms of chemical changes and microbial growth during the distribution and storage stages (Azlim et al., 2022; Müller & Schmid, 2019). The pH‐sensing indicators consist of two main parts: a solid base polymer and a pH‐sensitive dye (Pourjavaher et al., 2017; Xue Mei et al., 2020). Different synthetic and chemical dyes, such as methyl red, chlorine phenol, and bromothymol blue, are often used in pH‐sensing color change detectors (Dirpan et al., 2018). However, due to the toxicity and carcinogenicity of these chemical dyes and their adverse effects on human health and the environment, the use of these dyes is limited (Wu, Zhang, et al., 2021). Therefore, natural pigments obtained from plant sources are recommended as an alternative to chemical types (Bao et al., 2022). In previous studies, plant‐based natural pigments including curcumin (Taghinia et al., 2021), anthocyanins (Duan et al., 2021), chlorophyll (Chavoshizadeh et al., 2020), and betalains (Shabahang et al., 2022) have been used to develop smart packaging.

Anthocyanins are water‐soluble natural pigments belonging to the group of flavonoids. Depending on their different forms, they are responsible for the red, blue, and purple colors in many vegetables, flowers, and fruits (Oladzadabbasabadi et al., 2022). In general, the color of anthocyanins depends on the physical and chemical conditions of the environment, nature, type, and the number of sugar bound to anthocyanidin, chemical reaction with glycoside, nature, and acids bound to sugar, and the reaction of anthocyanin with other molecules (Kong et al., 2003). Also, the presence of methoxyl and hydroxyl groups affects the type and intensity of anthocyanin color (He & Giusti, 2010). In previous studies, various plant sources such as red cabbage (Chen et al., 2021), *Phyllanthus reticulatus* (Gasti et al., 2021), carrot (Ebrahimi Tirtashi et al., 2019), and purple sweet potato (Liu et al., 2021) have been used as the sources of anthocyanins in smart packaging films.

*Coleus scutellarioides* or *Solenostemon scutellarioides* belongs to the Lamiaceae family and is a prevalent ornamental plant (Sahu & Dewanjee, 2012) whose leaves are used in Indonesia as a traditional medicine to treat various diseases such as hemorrhoids, pain, inflammatory diseases, diarrhea, and diabetes (Zulfahmi & Solfan, 2010). In traditional Asian medicine, this plant is also used to treat sore throat, bronchitis, digestive problems, shortness of breath, and scorpion stings (Suva et al., 2015). Different phytochemical and functional compounds, such as flavonoids, volatile essential oils, alkaloids, polyphenols, and saponins, have been found in the leaves of this plant (Ahmad & Massi, 2014). The coleus leave*s* contain a remarkable amount of red anthocyanins (Logan et al., 2015). This herb has also reported significant antimicrobial and antioxidant activity (Aziz et al., 2020). Therefore, this research investigated *C. scutellarioides* anthocyanin extract as a natural source of anthocyanins to develop an intelligent packaging film based on fish gelatin biopolymer.

## 2 MATERIALS AND METHODS

### 2.1 Materials

Fish gelatin was purchased from the SIM supply company (Penang, Malaysia) and used without further modification. Fresh *C. scutellarioides* leaves were obtained from a local market (Damghan, Iran). All other chemicals and plasticizers used were of analytical grade and without further purification.

### 2.2 Extraction of anthocyanin extract from *C. scutellarioides*

*Coleus scutellarioides* (CS) powder was acquired according to Luchese et al. (2018) method with a slight modification. First, the CS leaves were washed and drained. Then, the leaves were freeze‐dried for 3 days, milled, and sieved. Next, the 200‐mesh powder was collected for the extraction of anthocyanins. Next, the CS powder (5 g) was added to a 100‐ml ethanol–water mixture (1:1 volume ratio) and stirred at 500 rpm for 1 h. After that, the solution was filtered through Whatman (No. 4) filter paper, and the obtained CS anthocyanin extract (CSAE) was kept in the dark at 4°C until film preparation.

### 2.3 Determination of total anthocyanin content

The total anthocyanin content of CSAE was measured using the pH differential method described by Giusti and Wrolstad (2001) and Sun et al. (2011). The CSAE were dissolved in potassium chloride buffer (KCl, 0.025 M, pH 1.0) and sodium acetate (CH_3_CO_2_Na∙3H_2_O, 0.4 M, pH 4.5) with a predetermined dilution factor. The absorbance was recorded at 510 and 700 nm against a blank cell containing distilled water. The absorbance (*A*) of the diluted sample was calculated as follows:

$A={\left(A_{\text{λvis}-\max}-A_{700\;\mathrm{nm}}\right)}_{\mathrm{pH}\;1.0}-{\left(A_{\text{λvis}-\max}-A_{700\;\mathrm{nm}}\right)}_{\mathrm{pH}\;4.5},$ where *A*_λvis‐max_ is the absorbance at *A*_λvis‐max_ 510 nm; *A*_700 nm_ is the absorbance at 700 nm. The monomeric anthocyanin pigment concentration in the original sample was calculated according to the following formula:

$\text{Anthocyanin content}\;\left(\mathrm{mg}/L\right)=\left(A\times \mathrm{MW}\times \mathrm{DF}\times 1000\right)/\left(ɛ\times 1\right),$ where *MW* is the molecular weight (449.2) of cyanidin‐3‐glucoside; *DF*, dilution factor (60); *ɛ*, molar absorptivity constant (29,600).

### 2.4 Preparation of gelatin/CSAE pH‐sensitive film

Fish gelatin/CSE intelligent films were prepared according to the method followed by Chi et al. (2020) with slight modifications. First, 40% (w/w, gelatin basis) of glycerol, used as a plasticizer, was blended into 100 ml of distilled water through stirring at 350 rpm until it reached 40–50°C. Then, 8.0 g of gelatin was added to the solution and stirred for 40 min at 60–70°C. The total anthocyanin content in the CSAE, determined by the pH differential method, was calculated as 39.27 ± 5.78 mg/L. CSAE was then added to the solution by 10, 20, and 30 ml of CSAE when the solution was cooled down to 40–50°C and stirred for 10 min. The film solution was cast into 16 × 16 cm and was dried at room temperature for 24 h and then continued drying in an oven at 40°C for 9 h. The film was prepared with 10, 20, and 30 ml of CSAE expressed as CSG1, CSG2, and CSG3, respectively, while the control film without CSE added is CSG0.

### 2.5 Film characterization

#### 2.5.1 The mechanical properties

The tensile strength (TS) and elongation at break (EAB) of the film strips were determined according to ASTM (2018) standard method D882‐18 with slight modifications by using the TA‐XT2TM Texture Analyzer (Stable Micro System). Before measurement of TS and EAB, the films strips (10 × 2 cm) were conditioned for 2 days at room temperature and 53% RH in a desiccator, and the thickness of each strip was measured at five randomly selected points with an ID‐C112XBS micrometer (Mitutoyo Corp.). Five replicates were carried out for each batch of film, and the best three that gave consistent results were chosen.

#### 2.5.2 Light transmittance

The barrier properties of gelatin films against ultraviolet (UV) and visible light were measured at selected wavelengths between 200 and 800 nm using a UV–Vis spectrophotometer (Model UV‐160A, Shimadzu Corp.). Each film sample was cut into rectangular pieces (40 × 10 mm) and placed on the inner side of a transparent plastic 10 mm cuvette. The transmittance was measured according to Parsaei et al. (2022).

#### 2.5.3 pH‐sensing evaluation

The color changes of pH indicator films were measured using a colorimeter (Model Minolta Spectrophotometer CM‐3500D) via the Choi et al. (2017) method with slight modifications. The colorimeter was first calibrated to standard black and white. Before the measurement, the pH indicator films were immersed in different buffer solutions, pH 4.21, 7, and 9, for 15 min. After removing the buffer solutions, each film was placed on a Petri dish, and its color was evaluated without drying. The L*, a*, and b* parameters were recorded to assess the color of the film. The color change (*∆*E) of the film exposed at different pH was calculated from an equation as follows (Ghanbarzadeh et al., 2010):

$$∆E=\sqrt{{\left(L_{i}^{*}-L^{*}\right)}^{2}+{\left(a_{i}^{*}-a^{*}\right)}^{2}+{\left(b_{i}^{*}-b^{*}\right)}^{2},}$$

where *L*_i_*, *a*_i_*, and *b*_i_* are color values of the standard pH indicator film (pH 7.0). If *∆E* > 3.5, clear difference in color can be noticed (Halasz & Csóka, 2018). Tests were carried out in triplicate.

#### 2.5.4 In vitro anthocyanin release study

The release rate of anthocyanin into the phosphate‐buffered saline solution at pH 6.5 and 37°C and stirred at 100 rpm was studied as described by Carlos‐Salazar and Valderrama‐Negrón (2017) with some modifications. For this purpose, the film sample (2.5 × 2.5 cm) was placed in a measuring cylinder containing 50 ml of food simulants. Of the solution, 2 ml was taken at predetermined time intervals (10 min for 200 min) and replaced with the same volume of fresh medium. The absorbance was read at 515.2 nm by a UV–Vis spectrophotometer (Shimadzu 1800, Shimadzu Corp.). A standard curve determined the released anthocyanin.

The release of anthocyanin from the films was estimated using the following equation:

$$\text{Release}\;\left(\%\right)\frac{M_{t}}{M_{0}}\times 100,$$

where *M*_t_ and *M*_0_ show the released anthocyanin at time *t* and the total anthocyanin loaded to the films, respectively.

#### 2.5.5 Application of the films as an indicator for freshness monitoring of rainbow fish

Gelatin/CSAE (CSG3) films were used for application as the packaging of fish to evaluate the freshness of fish during storage. About 60 g of fish samples were packed in polyethylene packs, and a piece of indicator (3 × 3 cm^2^) was placed inside the packs and kept at 25°C and 75% RH in an incubator. The total volatile basic nitrogen (TVB‐N) content of the fish was determined over storage time by stream distillation assay (Mirzapour‐Kouhdasht & Moosavi‐Nasab, 2020). Briefly, 10 g of minced samples were distilled and collected in a flask containing a few drops of methyl red and 2% boric acid. Then, the solution of the sample was titrated using sulfuric acid (0.1 N) until the appearance of purple color. TVB‐N content of samples was expressed as mg N/100 g of fish.

### 2.6 Statistical analysis

The statistical analyses were performed using a one‐way analysis of the variance (ANOVA), showing statistically different values. Meanwhile, the mean comparison was carried out by Duncan's test. The SPSS 26.0 software (IBM SPSS, Inc.) was used with differences at *p* < .05 considered significant.

## 3 RESULTS AND DISCUSSION

### 3.1 Total anthocyanin content of CSAE

Anthocyanins are the most significant subgroups of flavonoids and are known to have wide color ranging from red to violet. They can mainly be found in fruits and vegetables. As for this research, the anthocyanin was extracted from the CS leaves, and cyanidin‐3‐glucoside was the anthocyanin pigment content obtained in CS. The total anthocyanin content extracted from CS powder was calculated using the pH differential method. The total anthocyanin content in CS was 35.66 ± 3.14 mg/L. The anthocyanin content of the CS powder is thought to be higher than that obtained in this study because anthocyanin is quickly degraded, which might have contributed to the low results achieved. Anthocyanin was very sensitive to pH, light, and temperature. Since it is highly reactive, it easily degrades or reacts with other constituents in the film matrix to form colorless or brown‐colored compounds (Chaiyasut et al., 2016).

### 3.2 Mechanical properties of films

**FIGURE 1:**
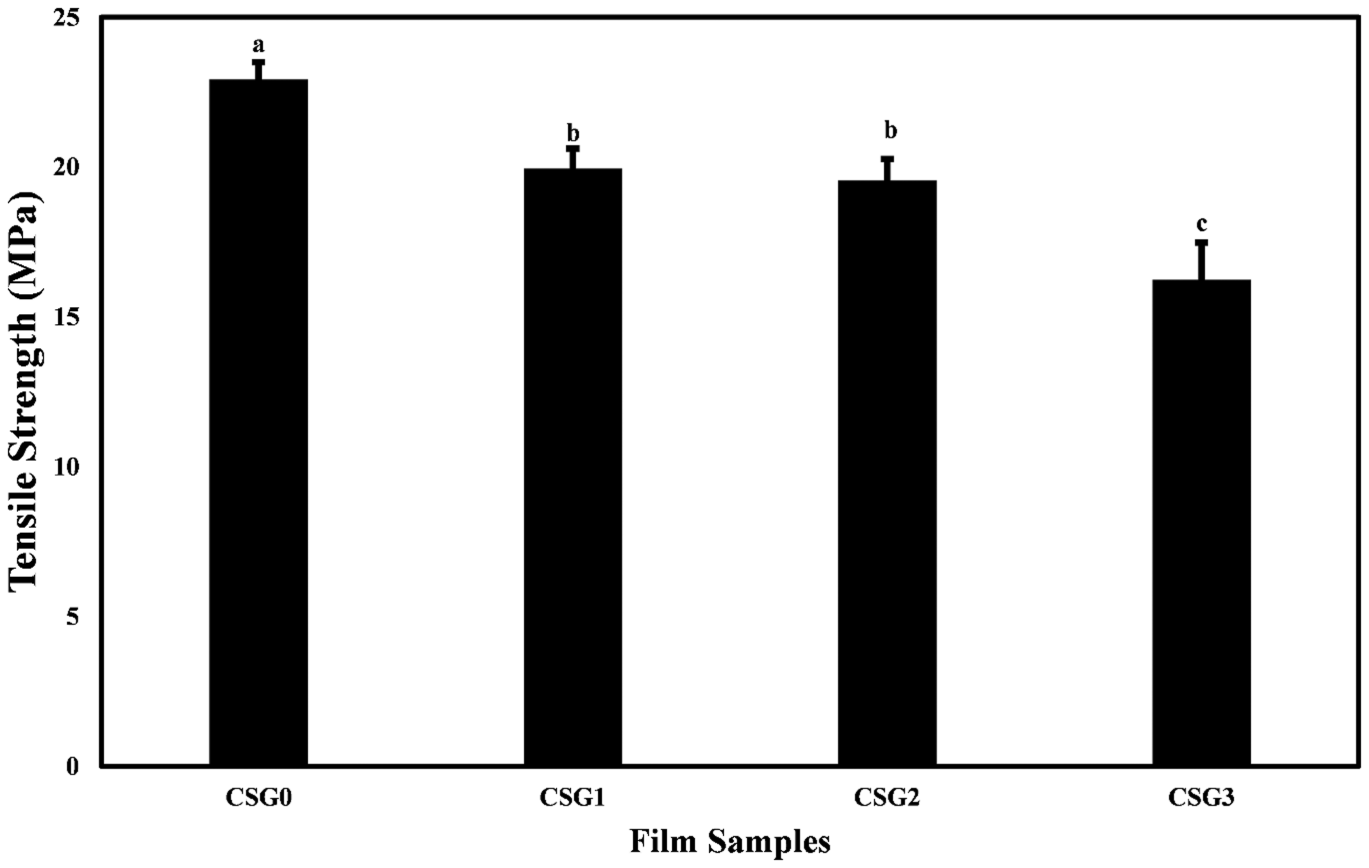
Effects of CSAE on the tensile strength (MPa) of fish gelatin‐based films. Data points with different letters indicate significant difference (*p* < .05) by Tukey's mean comparison test

**FIGURE 2:**
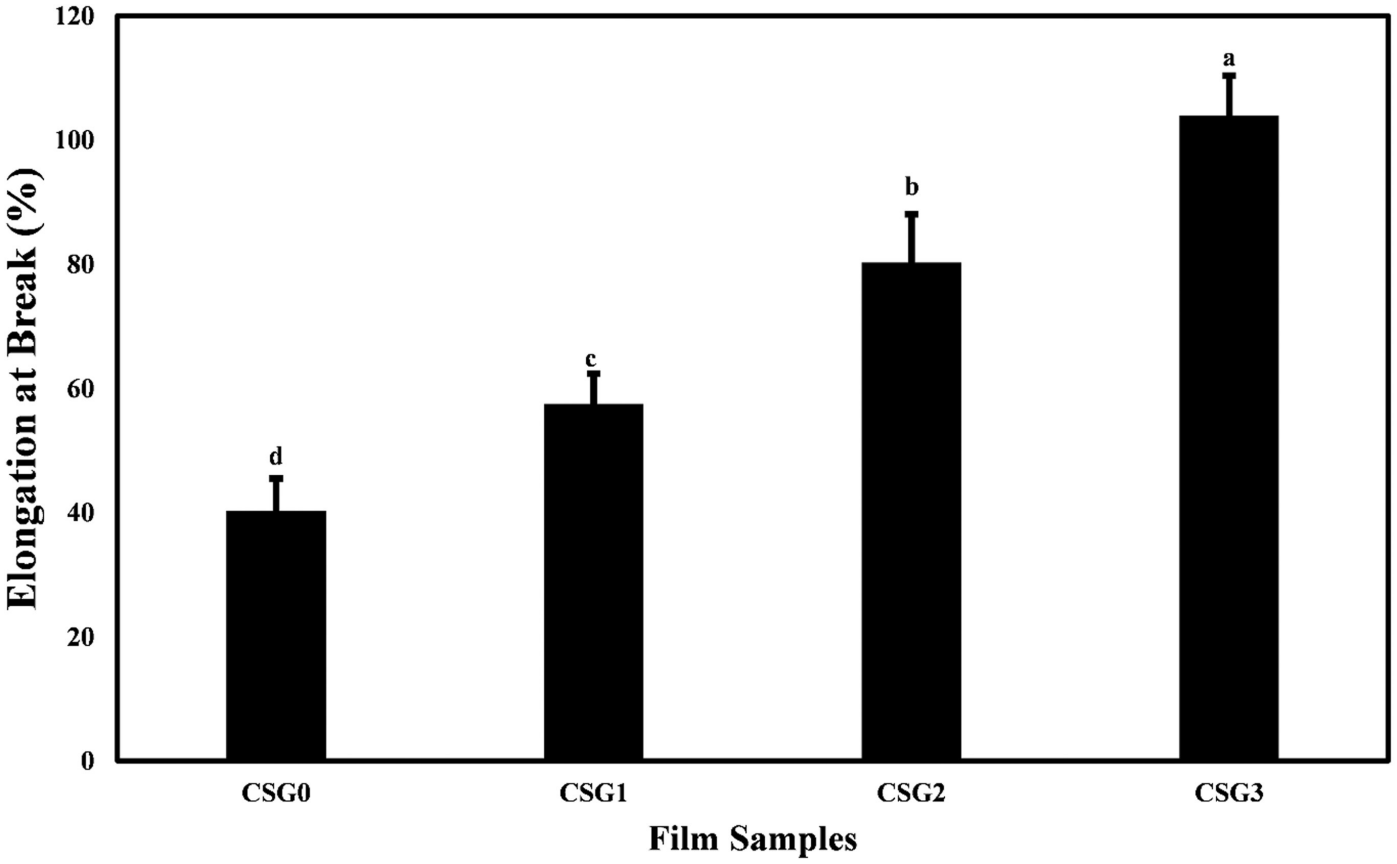
Effects of CSAE on the elongation at break (%) of fish gelatin‐based films. Data points with different letters indicate significant difference (*p* < .05) by Tukey's mean comparison test

The mechanical properties of the gelatin‐based films containing different levels of CSAE were expressed in terms of the tensile strength (TS) (pulling force per film cross‐sectional area required to break the film) and elongation at break (EAB) (the degree to which film can stretch before breaking) as shown in Figures 1 and 2, respectively. TS values for the films decreased significantly (*p* < .05) as the concentration of CSE added to the film increased. CSG0 showed the highest TS (22.917 ± 0.58 MPa) and CSG3 had the lowest TS (16.24 ± 1.24 MPa). The study by Prietto et al. (2017) obtained a similar trend: adding black bean anthocyanins reduced the film's tensile strength compared to the control film. According to their study, anthocyanin molecules could lower the TS value because the presence of anthocyanins can weaken the intermolecular interactions, which will affect the mechanical properties of the films.

The EAB value of the films obtained was inversely proportional to TS, where CSG0 has the lowest value (40.35%) and CSG3 has the highest value (104.01%). The study by Pourjavaher et al., (2017) obtained a similar trend: the decrease of the TS value and the increase of the EAB value in the film based on bacterial cellulose and anthocyanin from red cabbage extract. They reported that the phenolic compounds of red cabbage in the bacterial cellulose matrix probably act as a plasticizer and reduce the interactions among the macromolecules, leading to decreased TS and increased EAB value. This phenomenon also could be applied to the molecular interactions between fish gelatin and CS phenolic compounds. Also, covalent and hydrogen bonds can be established between anthocyanins and hydroxyl and amine groups of polypeptides in the gelatin structure. Film strength can be reduced by weakening protein–protein linkages (Li et al., 2014). In a study by Musso et al. (2019), the effect of anthocyanin plasticizer on the protein matrix of the film was reported. The incorporation of red cabbage anthocyanin extract reduced the TS and increased the EAB of gelatin films compared to the control film.

### 3.3 UV–Vis light transmittance

**FIGURE 3:**
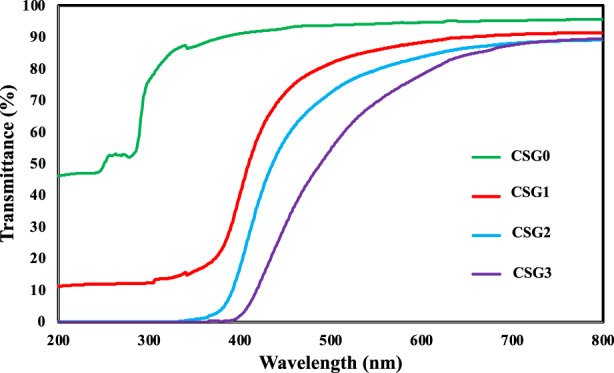
Effects of CSAE level on light transmittance (%) of fish gelatin‐based films

UV–Vis light barrier property of the film is very important for light‐sensitive food packaging. UV–Vis light can accelerate food degradation and oxidation, leading to nutrient destruction, color loss, and toxic substance formation (Peralta et al., 2019). UV–Vis spectrophotometry studied the transmittance (%T) in the UV–Vis range of the films, as shown in Figure 3. CSG1, CSG2, and CSG3 films containing CSAE showed significantly lower light transmittance in both UV and visible wavelength ranges than CSG0, suggesting the films with added CSE possessed higher UV–Vis light barrier ability than control films without CSE. Furthermore, the UV–Vis light barrier ability of CSG films was positively correlated with CSE content in the films. Similar to the findings from studies by Gutiérrez and Alvarez (2018), Luchese et al. (2018), and Yun et al. (2019) which they found that the UV–Vis light barrier property of anthocyanin‐rich starch films was elevated with the increase of the content of anthocyanins. According to Ashrafi et al. (2018), the aromatic rings in the anthocyanins can absorb UV–Vis light, contributing to the light barrier ability of the film. Therefore, the presence of anthocyanin by adding CSE into gelatin films can increase the lightweight barrier properties of the film. Improvement of UV–Vis blocking properties of gelatin/oxidized chitin nanocrystals was also reported due to the incorporation of black rice anthocyanins (Ge et al., 2020).

### 3.4 pH‐sensitive evaluation

Color properties are important in the appearance of pH indicator films. The color spectra of the films at different pH are shown in Table 1, showing the color of gelatin‐based films containing different levels of CSAE and the color change data. Incorporation of CSE has reduced the *L* values significantly (*p* < .05), except the CSG2 and CSG3 are insignificantly reduced. Given that *L* = 0 represents black and *L* = 100 represents white, a low L* value indicates film darkening. In addition, the values of a* and b* for the control film (CSG0) without CSAE added are the lowest compared with films that incorporated RPE. As the concentration of CSAE added increased, the values of a* decreased insignificantly and b* increased insignificantly, indicating the tendency of the film to show green and yellow coloration compared to the control film. Moreover, the total color difference increased when CSAE concentration increased, and higher ∆*E* values indicated increased coloration of the films. Thus, incorporating CSAE into the fish gelatin films led to a higher degree of darkening and enhanced green and yellow attributes. Other researchers have reported the darker and reddish colors of biopolymer‐based intelligent films due to anthocyanin extract (Uranga et al., 2018; Wang, Xia, et al., 2019; Wang, Yong, et al., 2019). Koosha and Hamedi (2019) indicated that the presence of black carrot anthocyanins in the chitosan/PVA films led to the change of a* amounts of films from negative to positive values, and the color of the produced films changed from green to red.

**TABLE 1:** The color parameters of fish gelatin‐based films containing different levels of CSAE immersed in different pH buffers

pH	Samples	L*	a*	b*	Δ*E*
–	CSG0	96.33 ± 0.04 a	5.07 ± 0.03 d	0.74 ± 0.28 c	–
	CSG1	93.47 ± 0.09 b	19.14 ± 0.07 c	12.83 ± 0.68 b	12.56 ± 0.37 c
	CSG2	92.43 ± 0.25 c	26.61 ± 0.07 b	16.66 ± 0.82 a	16.51 ± 0.49 b
	CSG3	92.13 ± 0.51 c	33.53 ± 0.18 a	17.87 ± 0.75 a	17.75 ± 0.44 a
pH 4.01	CSG0	4.74 ± 1.39 a	5.84 ± 0.14 d	−2.13 ± 0.11 a	–
	CSG1	5.09 ± 1.51 a	21.04 ± 0.09 c	−1.27 ± 0.14 a	1.22 ± 0.12 b
	CSG2	7.47 ± 1.38 ab	27.27 ± 0.17 b	−3.35 ± 0.28 a	4.62 ± 1.43 a
	CSG3	8.83 ± 0.69 b	36.22 ± 0.21 a	−1.84 ± 0.32 a	5.41 ± 1.27 a
pH 9.21	CSG0	6.03 ± 1.66 a	−0.76 ± 0.55 a	−3.59 ± 0.18 c	–
	CSG1	5.87 ± 1.07 a	−0.93 ± 0.36 a	−0.06 ± 0.22 b	1.07 ± 0.41 b
	CSG2	10.29 ± 1.37 b	−1.34 ± 0.24 a	0.79 ± 0.15 b	2.45 ± 0.08 a
	CSG3	12.87 ± 1.24 b	−1.23 ± 0.11 a	2.69 ± 0.14 a	0.94 ± 0.39 b

*Note*: Values represent mean (*n* = 3) ± *SD*. Different letters represent significant difference at 5% level of probability among sample, the samples in each column and each pH buffers. The upper rows at pH – are for intact films, not in the immersed state.

The CSG films were immersed in a pH buffer of 4.01 and 9.21 for 15 min to observe the color changes according to the pH value. The color of each film at the neutral pH (7.0) was used as a set point to establish the parameters for total variation in the film color. According to Halasz and Csóka (2018), the color changes of anthocyanins are due to the alterations in the molecular structure of anthocyanins in a wide range of pH. Under acidic conditions (pH 1–3), flavylium cation structure (purple and red colors) predominates. In the pH range of 4–5, pseudo‐base carbinol is the predominant form caused by the hydration of the molecule, resulting in a colorless indicator. Increasing the pH to 6–7 rendered the basic purple color structure of the quinoidal. This structure in an anionic form at pH 7–8 is blue. The central ring opens at a pH of 8–9, forming the yellow‐colored chalcone (Prietto et al., 2017).

Based on the result in Table 1, all the CSG films showed a low value of L*, indicating low brightness. At these pHs, an increase in the L* index of films was observed with increasing the level of CSAE. The results of chroma a* showed a negative relation to the pH: the higher the pH, the lower the a* value. CSG0 and CSG1 showed a very low intensity of red color at pH 4.01, while CSG2 and CSG3 showed a slightly green color. At pH 9.21, all the film showed a decreasing negative a* value, which indicates the low intensity of green color, similar to the study from Prietto et al. (2017). They observed that the film incorporated anthocyanin from black bean seed showed that the green color tends to dominate at higher pH. All the films at both pHs presented negative values of chroma b*, indicating a blue color, except the CSG2 and CSG3 at pH 9.21 showed positive values indicating a yellow color. Choi et al. (2017) and Peralta et al. (2019) observed the green color of the intelligent films containing the anthocyanin extract of sweet purple potato and marshmallow extracts at alkaline pH, respectively. Azlim et al. (2022) also demonstrated that the gelatin films containing dragon fruit skin extract were red to purple at an acidic pH, while the films had green‐blue color at the alkaline pH.

**FIGURE 4:**
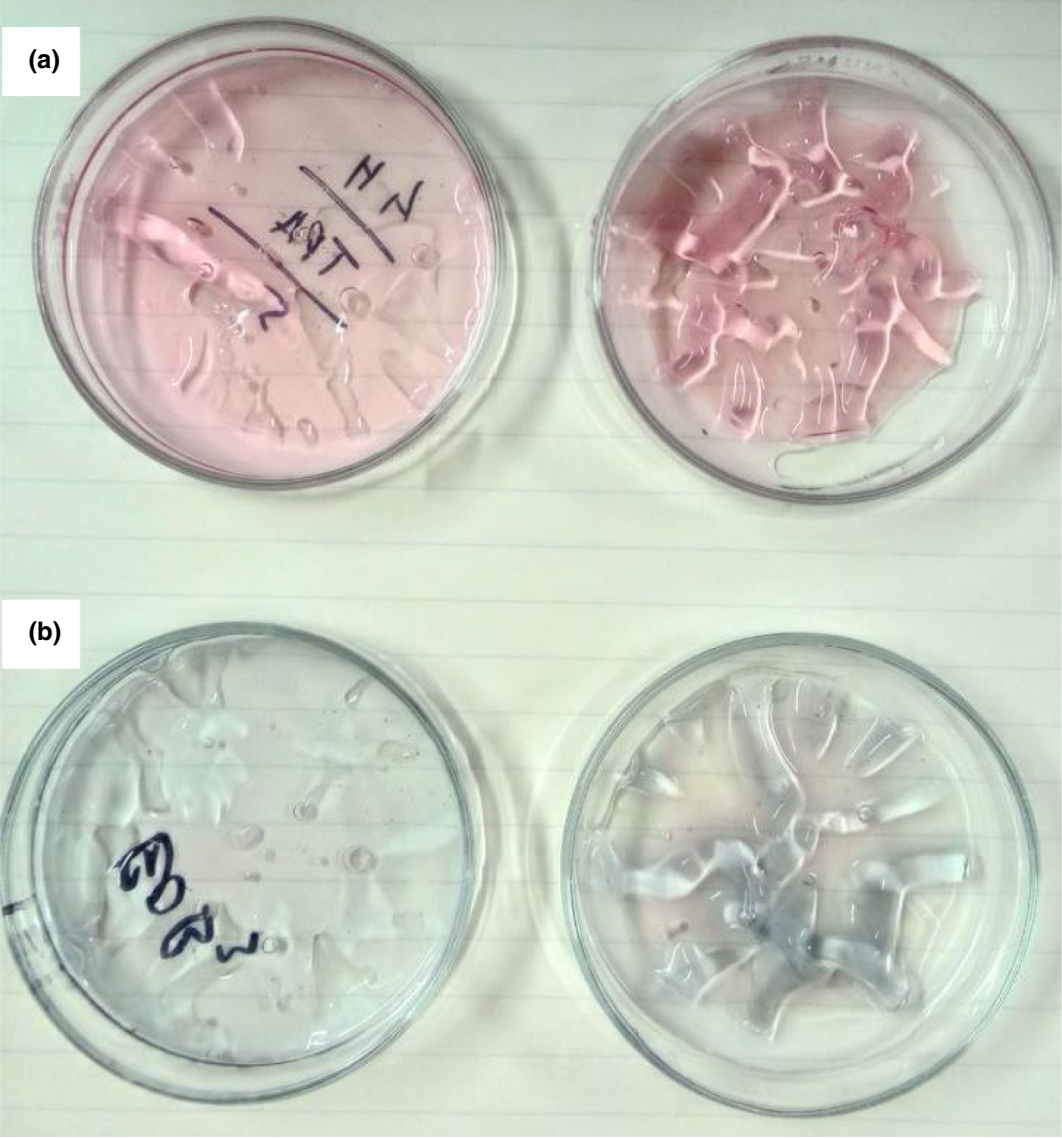
Color changes of the CSG3 films in both acidic and alkali solutions. (a) Acidic solution; (b) alkali solution

The color changes of the CSG3 films in acidic and alkaline solutions are shown in Figure 4. According to Prietto et al. (2017), ∆E* value of greater than 5 can be easily detected by the human eye, while ∆E* value above 12 implies an absolute difference in color which can be noticeable even by untrained panelists. All the films have ∆E* value below 12, indicating no distinct color changes between low and high pH. Furthermore, CSG1 and CSG2 films have limitations at pH 4.01 and 9.21, and CSG3 is present only at pH 9.21.

### 3.5 Evaluation of released anthocyanin from the films

**FIGURE 5:**
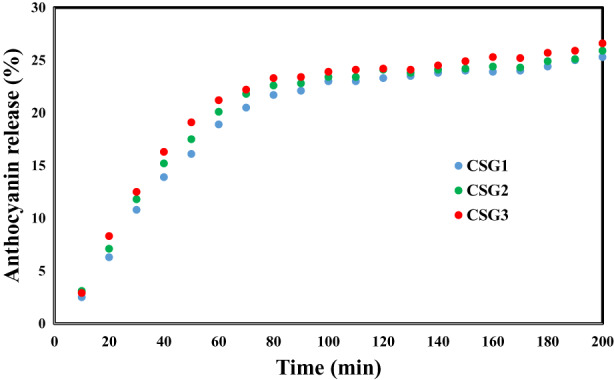
In vitro control release profile of anthocyanin from gelatin/CSE films

The films with different levels of CSAE are immersed in a simulated solution, and cumulative anthocyanin release is examined for 200 min. As shown in Figure 5, each release profile indicated an initial burst followed by a sustained release until it reached a plateau, revealing the controlled release of CSAE from gelatin films. It also can be observed that when the CSAE concentration is elevated, the release rate of anthocyanin to the medium increases considerably, attributed to the diffusion driving force from the loaded CSAE contents (Wang et al., 2017). Another reason for this result is that the films with more surface roughness provide better interaction with the release medium and improve the release of bioactive agents from the film (Carlos‐Salazar & Valderrama‐Negrón, 2017; Malikmammadov et al., 2018). Wu, Tsai, et al. (2021) and Wu, Zhang, et al. (2021) also found that in gellan gum‐based packaging films, the release of anthocyanins at pH >6 was controlled. Similarly, Wang, Xia, et al. (2019), Wang, Yong, et al. (2019) and Gasti et al. (2021) reported the gradual and controlled release of anthocyanin complexes from active biopolymer‐based films. Wei et al. (2017), in the development of active and intelligent films based on gellan gum containing sweet potato, also indicated that the films were able to control the release of anthocyanins at different pHs.

### 3.6 Monitoring the freshness of fish fillets with gelatin/CSAE films

**FIGURE 6:**
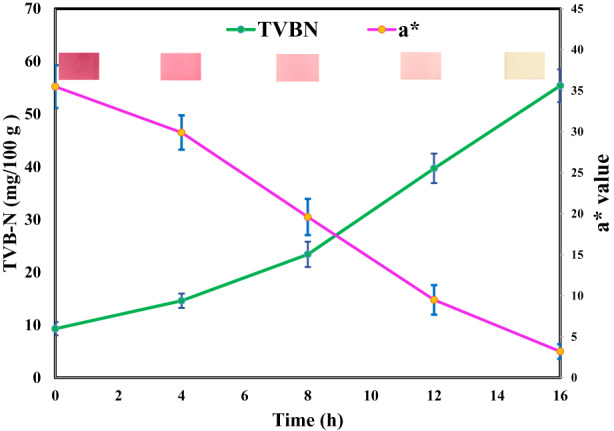
Variation of a* values of CSG3 films and TVB‐N of rainbow fish over storage time at room temperature

It has been reported that the primary reason for the spoilage of seafood products is microorganisms. TVB‐N is a product of microbial squalor, and its content in seafood is known as a chemical measure to evaluate the extent of spoilage. Because seafood and meat products are good protein sources and during the spoilage stage of these products proteins are destroyed by microorganisms and various nitrogenous compounds, such as amines and ammonia, are produced. The potential of gelatin/CSAE films to sense the spoilage of fish freshness was investigated in this work. The alteration of the TVB‐N content of fish at room temperature over storage time is shown in Figure 6. According to this figure, the initial TVB‐N level of fresh fish was 9.3 mg/100 g and increased to 55.4 mg/100 g with increasing storage time from 0 to 16 h. It also found that with increasing storage time, the film color altered from purple to colorless, demonstrating its ability to sense fish spoilage. The a* values of gelatin/CSAE films over storage time are also shown in Figure 6. Pearson's correlation test demonstrated a significant negative correlation between a* values of the films and the TVB‐N of the fish samples over storage time (Pearson's correlation = 0.976).

Zhang et al. (2019), in the development of biopolymer‐based smart packaging using *Roselle* anthocyanins, also found that the total color differences (Δ*E*) of films increased with increasing the TVB‐N values of pork during the 72‐day storage period at room temperature. Similarly, Zeng et al. (2019) observed that during the storage period and with the development of spoilage of fish samples and higher amounts of TVB‐N, a clear color change occurred in the films based on gelatin/PVA containing mulberry anthocyanin extracts. These researchers stated that due to the activity of microorganisms and the breakdown of carbohydrates, proteins, and fats, and the function of enzymes in fish samples, volatile alkaline compounds were produced, which led to an increase in the pH of the samples, as a result of which the color of the films containing anthocyanins changes. Furthermore, an increase in the TVB‐N values and Δ*E* of shrimp samples during the 36‐h storage period was reported by Bao et al. (2022). Other researchers have achieved similar results in the development of intelligent packaging films using anthocyanins to display the freshness of seafood products using TVB‐N changes in samples and the color changes of films (Ge et al., 2020; Jiang et al., 2020; Mohammadalinejhad et al., 2020).

## 4 CONCLUSION

This study successfully developed an intelligent fish gelatin indicator containing CSAE. The produced films indicated good color changes at different pHs. During the storage period of fish samples, by increasing the TVB‐N values, the indicator purple color was changed to colorless, demonstrating its ability to display fish spoilage. In summary, the fish gelatin‐based intelligent films containing CSAE could be used to display the freshness of seafood products and predict the shelf life of the products during storage time.

## FUNDING INFORMATION

The authors thank the fundamental research grant scheme (Grant Number FRGS/1/2022/STG05/USM/02/8) of the Ministry of Higher Education Malaysia for the financial support and coordination for the improvement of personals in Malaysian higher education sector.

## CONFLICT OF INTEREST

The authors declare no conflict of interest.

## ETHICAL STATEMENT

This study does not involve any human or animal testing.

## Data Availability

The data that support the findings of this study are available from the corresponding author upon reasonable request.
